# Selective Cytotoxicity of Ochratoxin A: Pro-Apoptotic Effects on Healthy Immune Cells Compared to Leukemia Cells

**DOI:** 10.3390/molecules30234497

**Published:** 2025-11-21

**Authors:** Magdalena Więckowska, Edyta Janik-Karpinska, Natalia Cichon, Ewelina Synowiec, Rafał Szelenberger, Maksymilian Stela, Marcin Podogrocki, Leslaw Gorniak, Tomasz Poplawski, Tomasz Sliwinski, Lukasz Krzowski, Michal Bijak

**Affiliations:** 1 Biohazard Prevention Centre, Faculty of Biology and Environmental Protection, University of Lodz, Pomorska 141/143, 90-236 Lodz, Poland; magdalena.wieckowska@biol.uni.lodz.pl (M.W.); edyta.janik@biol.uni.lodz.pl (E.J.-K.); natalia.cichon@biol.uni.lodz.pl (N.C.); rafal.szelenberger@biol.uni.lodz.pl (R.S.); maksymilian.stela@biol.uni.lodz.pl (M.S.); marcin.podogrocki@biol.uni.lodz.pl (M.P.); leslaw.gorniak@biol.uni.lodz.pl (L.G.); 2 Department of Molecular Genetics, Faculty of Biology and Environmental Protection, University of Lodz, Pomorska 141/143, 90-236 Lodz, Poland; ewelina.synowiec@biol.uni.lodz.pl (E.S.); tomasz.sliwinski@biol.uni.lodz.pl (T.S.); 3 Department of Microbiology and Pharmaceutical Biochemistry, Medical University of Lodz, Mazowiecka 5, 92-215 Lodz, Poland; tomasz.poplawski@umed.lodz.pl; 4 Biomedical Engineering Centre, Institute of Optoelectronics, Military University of Technology, Kaliskiego 2, 00-908 Warsaw, Poland; lukasz.krzowski@wat.edu.pl

**Keywords:** ochratoxin A, mycotoxin, leukemia, immune cells, hematopoietic toxicity, hematological disorders

## Abstract

Ochratoxin A (OTA) is a widespread mycotoxin with documented nephrotoxic, hepatotoxic, immunotoxic, and carcinogenic effects, while its role in hematological malignancies and immune cells remains insufficiently defined. This study examined the cytotoxic and pro-apoptotic OTA activity in three human leukemia cell lines (CCRF-CEM, K-562, HL-60) and in peripheral blood mononuclear cells (PBMCs) from healthy donors. Cell viability was determined using 3-(4,5-dimethylthiazol-2-yl)-2,5-diphenyl tetrazolium bromide (MTT) and trypan blue assays, mitochondrial membrane potential (ΔΨM) was assessed with JC-1 dye, caspase-3/7 activity was measured by flow cytometry, and the expression of apoptosis-related genes was analyzed by RT-qPCR. OTA did not significantly affect viability, mitochondrial function, or caspase activity in leukemia cell lines, suggesting relative resistance to OTA-induced apoptosis. In contrast, PBMCs exhibited clear dose- and time-dependent sensitivity, manifested by reduced viability, ΔΨM, caspase-3/7 activation, and transcriptional changes consistent with intrinsic apoptosis, including decreased BCL-2 (anti-apoptotic) and increased BAX (pro-apoptotic), APAF1 (apoptosome component), CASP3, and CASP9 (executioner and initiator caspases) expression. These findings demonstrate that OTA selectively targets healthy immune cells rather than leukemia cells, highlighting its pronounced immunotoxic risk and the importance of caution when considering its effect in a hematological context. Although limited to in vitro models, this study underscores the necessity of further research to clarify the molecular basis of differential OTA sensitivity and its contribution to immunosuppression and hematological disease.

## 1. Introduction

Ochratoxins are a group of mycotoxins produced by fungi belonging to the genera *Aspergillus* and *Penicillium*. Among them, ochratoxin A (OTA) is considered the most toxic [1]. Its toxicological relevance arises from both its widespread occurrence in food products, such as cereals, coffee, dried fruits, wine, and cocoa products [2,3,4,5,6,7,8,9,10,11,12,13] and from a broad spectrum of harmful effects, including nephrotoxicity, hepatotoxicity, immunotoxicity, genotoxicity, teratogenicity, neurotoxicity, and carcinogenicity [14].

Following ingestion, OTA is absorbed through the gastrointestinal tract into the systemic circulation, where it has a strong affinity for serum proteins, particularly albumin. Its persistence and systemic distribution are facilitated by enterohepatic recirculation, which enables repeated reabsorption of OTA from the intestinal lumen into the bloodstream. Moreover, reabsorption occurs within both the proximal and distal renal tubules, further contributing to its retention. Consequently, OTA accumulates in the blood and various organs [1]. Multiple studies have demonstrated the occurrence of OTA in blood samples, providing evidence of its systemic distribution. Zhu et al. [15] reported that following a single oral dose of OTA (500 µg/kg) in lactating sows, peak plasma concentrations (920.25 ± 88.46 µg/L) were detected nine hours post-administration, with an elimination half-life of 78.47 ± 7.68 h [15]. In a related study, donkeys received a single oral dose of OTA (2500 µg/kg body weight), and plasma monitoring revealed a peak concentration of 10.34 µg/mL at 12 h post-administration [16].

The distribution of OTA within the body suggests a pronounced effect on the circulatory system. Evidence indicates that OTA exerts a toxic impact on blood cells, hematological function, and potent immunosuppressive effect. OTA induces apoptosis in human lymphoid T cell line in a time- and dose-dependent manner via caspase activation and mitochondrial dysfunction. Moreover, OTA decreases Bcl-x(L) protein levels, which only highlights the pro-apoptotic effect [17]. OTA disrupts humoral and cellular immunity by impairing bone marrow hematopoiesis and reducing lymphoid and myeloid progenitors, weakening overall immune function. This compromised immune surveillance, including decreased NK cell activity, may increase susceptibility to cancers [18].

As reported by Xu et al. [19], OTA induces dose-dependent immune toxicity in porcine alveolar macrophages (PAMs, 3D4/21), including decreased cell viability, increased apoptosis, elevated lactate dehydrogenase release, altered B-cell lymphoma 2-BCL2-associated X protein (Bcl-2/Bax) ratio, and upregulation of pro-inflammatory cytokines. This effect is mediated by reactive oxygen species (ROS)-dependent activation of toll-like receptor 4/myeloid differentiation primary response 88 (TLR4/MyD88), and downstream extracellular signal-regulated kinases 1 and 2 (ERK1/2), p38, and nuclear factor kappa-light-chain-enhancer of activated B cells (NF-κB) signaling [19]. Exposure to OTA has been shown to modulate immune responses and promote pro-inflammatory signaling. In experimental models, OTA increased the production of cytokines such as interleukins (IL-1β, IL-6) and tumor necrosis factor-alpha (TNF-α) in macrophages and enhanced the differentiation of naïve T cells into Th1 and Th17 subsets. These effects were accompanied by upregulation of Signal Transducers and Activators of Transcription (Stat1, Stat3, and Stat4) and suppression of Suppressor of cytokine signaling (Socs)-mediated feedback inhibition, indicating that OTA can stimulate immune activation and dysregulate normal immune control mechanisms, highlighting the potential of OTA to contribute to immune dysfunction and inflammation [20]. Moreover, studies in isolated perfused rat livers demonstrate that OTA can directly stimulate cytokine release from hepatic tissue, including TNF-α, and modulate the response to other mycotoxins and bacterial lipopolysaccharide, which underscores the broader immunomodulatory potential of OTA [21].

TNF-α and IL-6 are central mediators of communication between the immune and skeletal systems. TNF-α inhibits osteoblast activity at certain stages of differentiation while promoting osteoclast proliferation and differentiation, thereby contributing to bone resorption. Additionally, IL-6 regulates the activity of osteoblasts, osteoclasts, and osteocytes through complex mechanisms, exhibiting dual effects on bone metabolism. Dysregulation of both has been linked to immune-mediated bone disorders [22].

OTA has been associated with bone damage, manifested by deformations, reduced mineralization, neurodegenerative changes, enhanced resorption, and impaired growth [23]. In an in vivo study, mice receiving cumulative doses of 20–40 mg/kg OTA exhibited suppression of bone marrow granulocyte-macrophage progenitors, which gradually recovered within weeks, depending on the dose. Furthermore, researchers concluded that exposure to whole-body irradiation in OTA-treated mice resulted in delayed recovery of bone marrow and peripheral blood parameters compared to controls, suggesting that residual OTA toxicity increases the susceptibility of the hematopoietic system to secondary insults [24].

In a study conducted by Al-Redha et al. [25], a significant correlation between OTA presence in blood and cancer risk was identified. The study, which included oncology patients and healthy volunteers as controls, demonstrated that OTA was detectable in 60% of cancer patients in comparison to 12% of the control group [25]. Moreover, OTA was detected more frequently and at higher levels in the blood of individuals with Balkan Endemic Nephropathy or urinary tract tumors compared to healthy people from endemic and control regions, supporting its potential role in the etiology of both diseases [26]. Leukemia, a malignancy of the blood and bone marrow [27], can be influenced by various external factors, e.g., those derived from diet or environmental pollution [28].

Taking all this into account, including the ubiquity of OTA, its distribution in the body, demonstrated toxicity toward blood components and bones, immunotoxicity, and its potential carcinogenicity, it is essential to better understand its effect on hematopoietic and immune cells. Therefore, this study aimed to compare cytotoxic and pro-apoptotic activity of OTA in human leukemia cell lines and immune cells from healthy donors to elucidate the mechanism underlying its selective toxicity. The goal was to provide further insight into its toxicological impact and possible role in hematological disorders.

## 2. Results

### 2.1. Cell Viability

**Figure 1:**
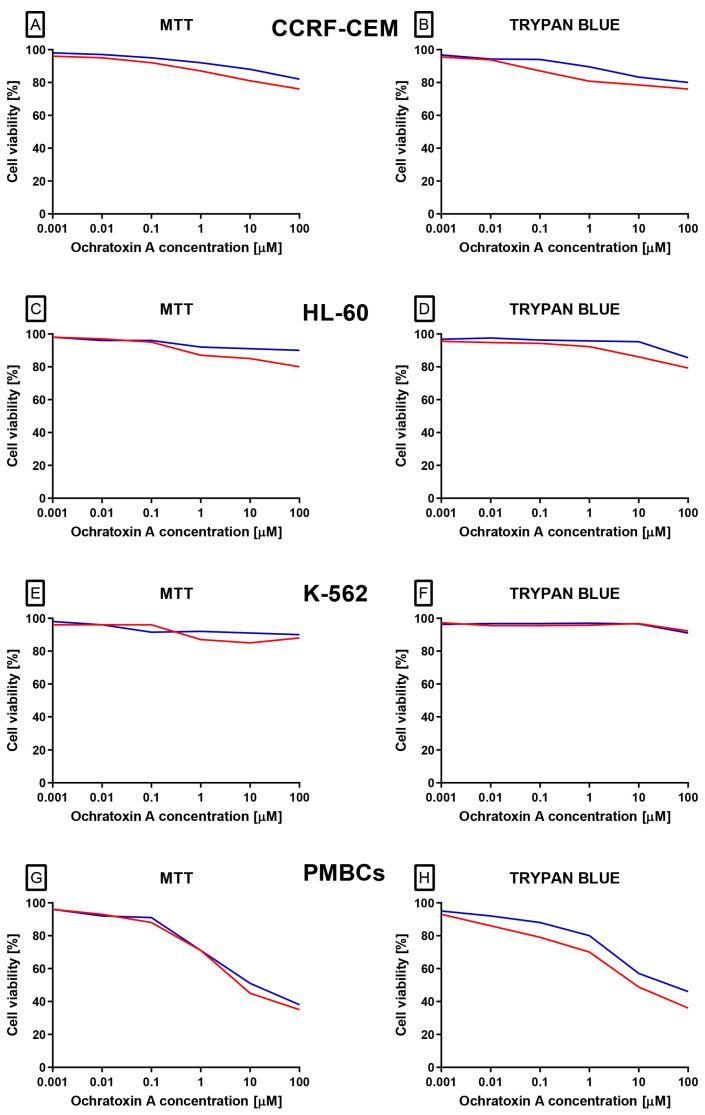
The effect of OTA (in range concentration range 0.001 to 100 µM) on CCRF-CEM (**A**,**B**), K-562 (**C**,**D**), HL-60 (**E**,**F**) and PBMCs (**G**,**H**) cells. Cell viability was estimated by using MTT and trypan blue methods. The data represents cell viability curves obtained from 4 independent measurements (*n* = 4). Viability is expressed as % relative to untreated control, which was set as 100%. The blue lines represent 24 h incubation, whereas the red lines represent 48 h incubation.

The first and crucial stage of our analysis was to analyze effect of OTA on different white blood cells origin cell lines the acute lymphoblastic leukemia line CCRF-CEM (CCL-119), the chronic myelogenous leukemia line K-562 (CCL-243), and the promyelocytic leukemia line HL-60. Additionally, analysis was also performed on Peripheral blood mononuclear cells (PBMCs) collected from healthy donors. Based on two independent methods: the trypan blue and 3-(4,5-dimethylthiazol-2-yl)-2,5-diphenyl tetrazolium bromide (MTT) assays, it was demonstrated (Figure 1A–H) that OTA did not cause cytotoxic effect on leukemia cell lines: CCRF-CEM, K-562 and HL-60. In case of PBMCs a dose-dependent cytotoxic effect of the compound in both methods was observed. Cytotoxicity curves illustrate this effect have been presented on Figure 1G,H.

### 2.2. Mitochondrial Membrane Potential (ΔΨM)

**Figure 2:**
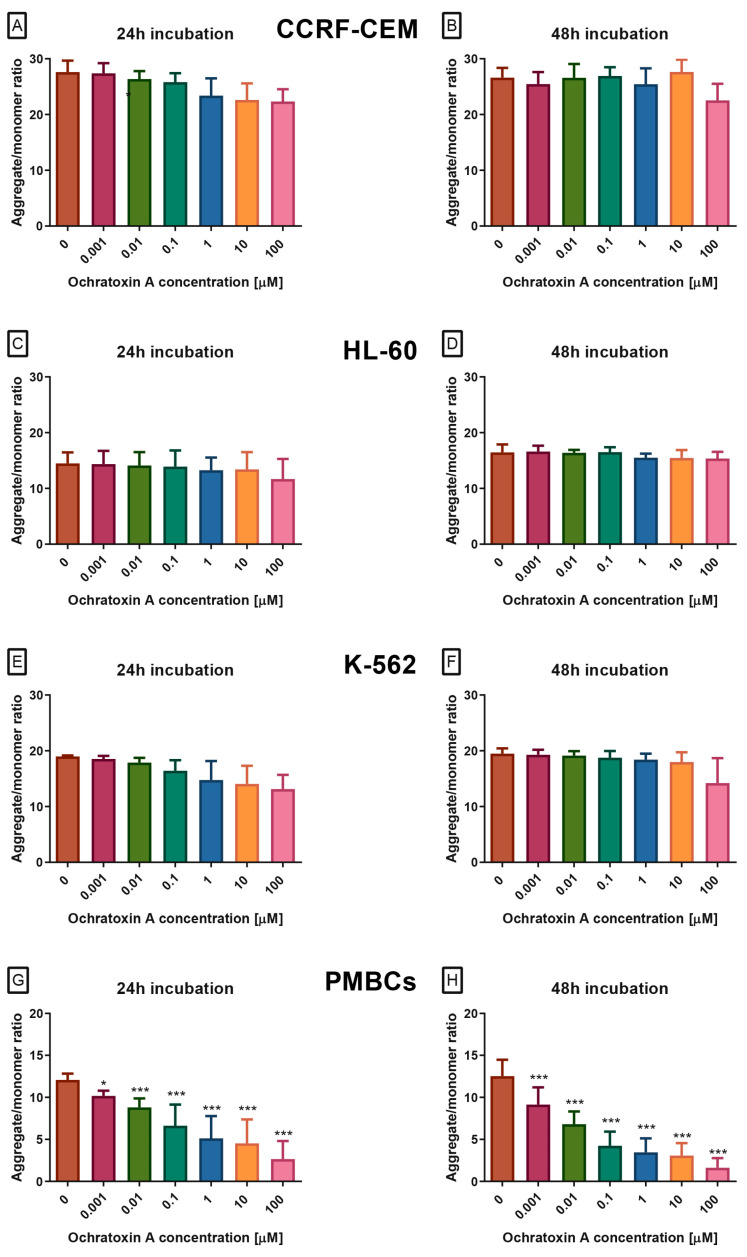
The Effect of OTA on mitochondrial membrane potential (ΔΨM) in CCRF-CEM (**A**,**B**), K-562 (**C**,**D**), HL-60 (**E**,**F**) and PBMCs (**G**,**H**) cells. ΔΨM was assessed using JC-1 dye and is expressed as the ratio of JC-1 aggregates (red fluorescence) to monomers (green fluorescence). A decrease in this ratio reflects mitochondrial depolarization, an early indicator of intrinsic apoptosis. Data are presented as mean ± SD (*n* = 4). * *p* < 0.05; *** *p* < 0.001 compared to the control. Caspase-3/7 activity.

To gain a deeper understanding of the toxic potential of OTA in human leukemia cells and healthy peripheral blood mononuclear cells, ΔΨM was evaluated using the JC-1 fluorescent probe, which is not influenced by mitochondrial morphology, size, or density. In leukemia cell lines CCRF-CEM, K-562, and HL-60, OTA exposure did not significantly alter the JC-1 aggregate-to-monomer fluorescence ratio (Figure 2A–F), indicating no measurable effect on ΔΨM. In contrast, PBMCs displayed a clear, dose- and time-dependent reduction in this ratio (Figure 2G,H), reflecting mitochondrial depolarization, an early event in intrinsic apoptosis. This decrease was evident across the full concentration range tested (0.1–100 µM) and reached statistical significance at all concentrations and incubation periods.

**Figure 3:**
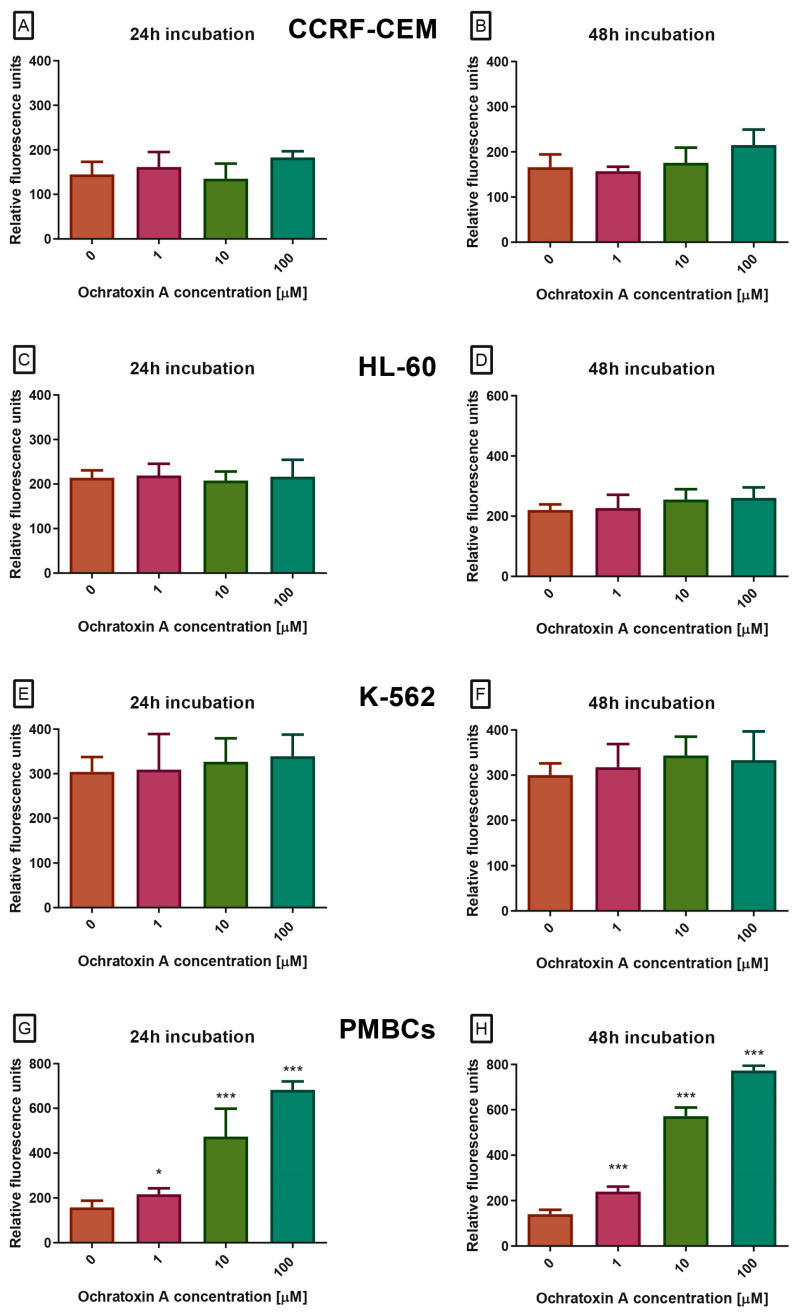
The Effect of OTA on caspases 3/7 pathway in CCRF-CEM (**A**,**B**), K-562 (**C**,**D**), HL-60 (**E**,**F**) and PBMCs (**G**,**H**) cells. Caspase proteolytic activity was measured by fluorescence of 485 nm/538 nm after 24 h and 48 h of incubation. Data are presented as mean ± SD (*n* = 4). * *p* < 0.05; *** *p* < 0.001 compared to the control.

At the next stage, caspase activity in cells exposed to OTA was examined. Caspases are cysteine proteases that play a central role in apoptosis, controlling both its initiation and execution phases. Among them, caspases-3 and -7 act as the main executioners, cleaving numerous cellular substrates and driving the characteristic morphological and biochemical features of apoptotic cell death. Because of this function, caspase-3/7 activity is widely recognized as a hallmark of the execution phase of programmed cell death. In our study, OTA at concentrations of 0.001–100 µM, after 24 or 48 h, did not alter caspase activity in the CCRF-CEM, K-562, or HL-60 cell lines (Figure 3A–F). In contrast, PBMCs treated with OTA showed a dose- and time-dependent increase in caspase-3/7 proteolytic activity, measured as green fluorescence (excitation/emission 485/538 nm, Figure 3G,H). All tested concentrations produced a statistically significant effect.

**Figure 4:**
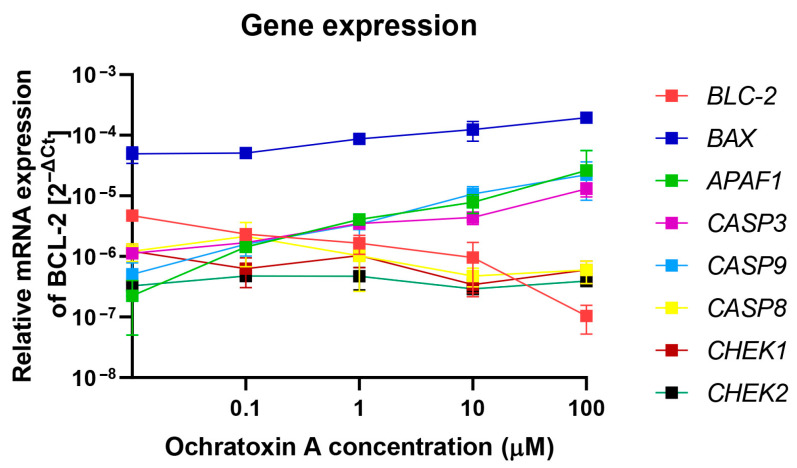
The effect of OTA on the expression of selected genes in PBMCs was measured at the mRNA level by RT-qPCR. Gene expression levels were normalized to 18S rRNA and expressed as 2^−ΔCt^ relative to untreated control cells, with the control set to 1. Data are presented as mean ± SD (*n* = 4).

To confirm activation of apoptosis process in PMBCs by OTA molecular analysis of the expression of key genes involved in apoptosis was performed. The analysis of mRNA levels in PBMCs treated by OTA were assessed using the RT-qPCR method. The purity of isolated RNA samples measured as the A260/280 ratio was in the range of 1.8–2. Three groups of genes were analyzed. The first category consisted of genes involved in the intrinsic apoptotic pathway, including BCL-2 (anti-apoptotic protein that inhibits mitochondrial cytochrome C release), BAX (pro-apoptotic protein promoting mitochondrial outer membrane permeabilization), and APAF1 (apoptotic protease activating factor 1, a key adaptor protein required for apopto-some formation and caspase-9 activation). The second category covered genes encoding caspases—CASP3, CASP8, and CASP9. The third category comprised genes associated with cell-cycle control, namely CHEK1 and CHEK2. The results of this analysis has been demonstrated at Figure 4. Within the intrinsic pathway gene set, BCL-2 expression declined in a dose-dependent manner, while BAX and APAF1 were upregulated, consistent with activation of the mitochondrial apoptotic pathway. For the caspase genes, increased expression was detected for CASP3 and CASP9, whereas CASP8 showed no significant change. Likewise, transcript levels of the cell-cycle checkpoint genes CHEK1 and CHEK2, which encode serine/threonine kinases involved in DNA damage sensing and G1/S or G2/M cell-cycle arrest, remained unaltered. These genes were included as controls to assess whether OTA induces DNA-damage–mediated cell-cycle checkpoints, in addition to triggering apoptosis.

**Figure 5:**
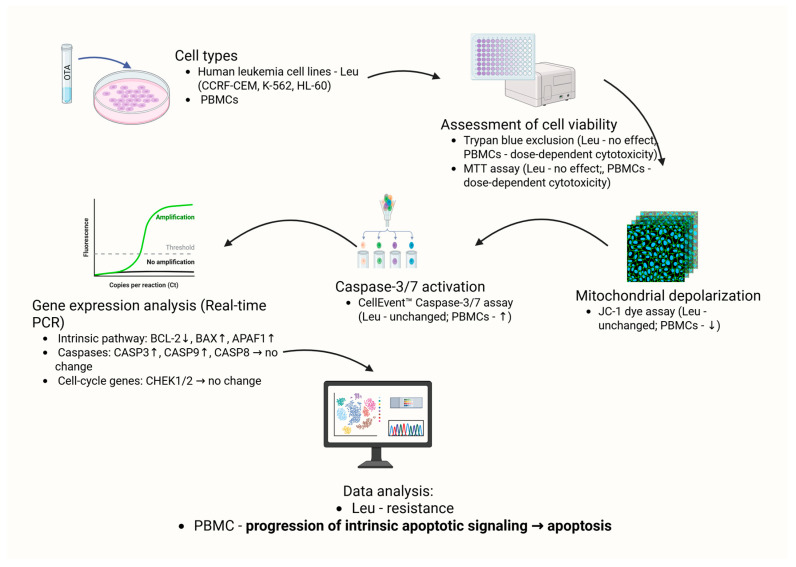
Schematic overview of OTA-induced effects in blood cells: leukemia cell lines (CCRF-CEM, K-562, HL-60) and PBMCs. Arrows indicate observed changes in cell viability (Trypan Blue, MTT), mitochondrial membrane potential (JC-1), caspase-3/7 activity (CellEvent™), and apoptosis-related gene expression (qPCR). Dose- and time-dependent effects were observed in PBMCs, whereas leukemia cell lines showed no significant alterations. Created in BioRender. Bijak, M. (2025) https://BioRender.com/nzj33t8 (accessed on 31 October 2025).

A schematic summary of the effects of OTA on leukemia cell lines and healthy PBMCS is presented in Figure 5. The diagram integrates findings obtained from cytotoxicity assays, mitochondrial membrane potential measurements, caspase activation analyses, and gene expression profiling.

## 3. Discussion

Apoptosis is initiated by disruption of mitochondrial integrity. Under physiological conditions, the mitochondrial permeability transition pores remain closed. Their opening results in the loss of mitochondrial membrane potential (ΔΨM), depolarization, the release of glutathione and Ca^2+^ ions into the cytoplasm, and a decrease in intracellular pH. Consequently, pro-apoptotic proteins previously confined to the mitochondria are released into the cytoplasm, thereby triggering the apoptotic cascade [29,30,31,32]. In our study, OTA exposure induced a dose- and time-dependent decrease in ΔΨM in PBMCs, whereas leukemia cell lines showed no significant changes, consistent with selective induction of the intrinsic apoptotic pathway. OTA was found to induce dose- and time-dependent apoptotic changes in PBMCs, including the loss of ΔΨm, an increase in caspase-3/7 activity, and modulation of pro- and anti-apoptotic gene expression (a decrease in BCL-2 transcript accompanied by an increase in BAX, APAF1, CASP3, and CASP9). Importantly, these effects displayed a clear time-dependent pattern, with early mitochondrial depolarization preceding caspase activation and transcriptional modulation. This sequence suggests a stepwise progression of apoptotic signaling in PBMCs following OTA exposure. The upregulation of APAF1 observed in PBMCs provides additional support for the involvement of the intrinsic apoptotic pathway. Increased APAF1 expression is indicative of apoptosome assembly, a crucial step linking mitochondrial cytochrome C release to the activation of caspase-9 and subsequent executioner caspases.

Interestingly, despite OTA’s well-documented genotoxic potential [17,33], no significant changes in CHEK1 and CHEK2 expression were observed in PBMCs. Liu et al. demonstrated that OTA induces oxidative DNA damage and G1 phase arrest, processes typically associated with activation of checkpoint kinases [34]. The absence of such changes in our study may reflect the timing of sample collection—transient activation of checkpoint pathways could have occurred earlier than the 24–48 h windows examined [35,36]—or may indicate that the observed apoptotic response proceeds predominantly via p53-dependent mitochondrial signaling rather than through canonical Chk1/Chk2-mediated cell-cycle arrest [37].

These findings are supported by previous reports showing that OTA induces apoptosis in human PBMCs via the intrinsic mitochondrial pathway. Assaf et al. [17] demonstrated significant increase in the proportion of apoptotic cells in a dose- and time-dependent manner. Caspase activation was observed concurrently with a reduction in the levels of the anti-apoptotic protein Bcl-xL, indicating the involvement of the mitochondrial pathway. Importantly, apoptosis was inhibited by the non-selective caspase inhibitor z-VAD-fmk, confirming the dependence of the process on caspase activity [17]. Similarly, Liu et al. [34] reported that low concentrations of OTA in human PBMCs induced the formation of ROS, the accumulation of oxidative DNA damage (8-OHdG), and cell cycle arrest in the G1 phase. These processes were accompanied by the activation of the apoptotic pathway, which was interpreted as a consequence of the initiation of the mitochondrial cell death mechanism [34]. Likewise, Zhang et al. [38] showed OTA-induced the loss of ΔΨm, the activation of caspase-9 and caspase-3, and DNA fragmentation, consistent with the classical intrinsic apoptotic pathway [38]. Importantly, Yang et al. [37] performed a transcriptomic analysis of cells treated with OTA and identified a range of genes involved in the regulation of apoptosis, including increased expression of CASP3 and p53AIP1, as well as the activation of pathways associated with the oxidative stress response. These findings are consistent with our observations of increased CASP3 and CASP9 transcripts and decreased BCL-2 expression [37]. These effects were consistently confirmed by MTT and trypan blue assays, indicating that PBMCs are genuinely sensitive to OTA, whereas leukemia cell lines remained largely resistant under the same conditions.

In contrast to PBMCs, no significant changes in viability, ΔΨm, or caspase activity were observed in the leukemia cell lines CCRF-CEM, K-562, and HL-60 following OTA exposure, indicating relative resistance to its pro-apoptotic effects. Similar observations regarding differential sensitivity among various cell lines have been reported by other researchers. Skrzydlewski et al. [39], in a literature review, highlighted that the cellular response to OTA is strongly dependent on molecular status, particularly on the levels of anti-apoptotic Bcl-2 family proteins, mutations in the TP53 gene, and the activity of detoxification mechanisms [39]. Cancer cells often exhibit elevated expression of Bcl-2 or Mcl-1, which stabilize the mitochondrial membrane and inhibit cytochrome C release, thereby blocking caspase-9 activation and the subsequent apoptotic cascade [40,41,42,43]. Similar mechanisms underlying the resistance of cancer cells to OTA-induced apoptosis have been described by Erceg et al. [44]. In their study of hepatocytes and stem cells, OTA was shown to induce caspase activation and apoptotic cell death; however, this effect was markedly attenuated in models exhibiting high expression of anti-apoptotic proteins [44]. In leukemia cell lines such as K-562 and HL-60, elevated Bcl-2 expression has been repeatedly documented in the literature and associated with chemoresistance [45,46,47,48,49], which may also explain the lack of response observed in our study. These observations are in line with recent findings by Juan-García et al. [50] assessed the individual and combined cytotoxic effects of OTA, beauvericin (BEA), and enniatin B (ENN B) in PBMCs, HL-60 leukemia cells, and MDA-MB-231 breast cancer cells. OTA alone was found to exert the greatest cytotoxicity in PBMCs (IC_50_ = 0.5 μM), whereas ENN B exhibited higher potency in HL-60 and MDA-MB-231 cells. Importantly, binary combinations demonstrated cell type–specific effects, with ENN B + OTA showing the highest cytotoxicity in HL-60 and MDA-MB-231, while BEA + OTA was most toxic in PBMCs. The triple combination was highly cytotoxic for PBMCs compared with the cancer cell lines. Overall, PBMCs were identified as the most sensitive cell type, and the presence of OTA consistently contributed to synergistic cytotoxicity [50].

Another factor potentially contributing to leukemia cell resistance is the status of the TP53 gene. Many leukemia cell lines, including K-562, harbor mutations or deletions in p53, impairing oxidative stress and DNA damage response pathways [51,52,53]. Liu et al. [34] demonstrated that, in PBMCs, OTA induces the accumulation of ROS and oxidative DNA damage, resulting in p53 activation and the initiation of the intrinsic apoptotic pathway [34], a mechanism likely inactive in leukemia cell lines, explaining their relative resistance [54].

In our study, OTA did not induce an increase in necrosis markers, as assessed by SYTOX™ AADvanced™ staining, regardless of the type of cells analyzed. This is an important observation, as the literature indicates that, under certain conditions, OTA can lead to necrotic cell death. Kupski et al. [55] showed that OTA induces necrotic cell death in human neutrophils, associated with increased intracellular calcium concentration, enhanced respiratory burst, a decrease in ATP levels, and disruption of ΔΨm. Moreover, the authors noted that this effect was abolished when OTA was converted into a less toxic metabolite, ochratoxin α [55]. In turn, Xie et al. [56] presented evidence suggesting that OTA may initiate so-called PANoptosis in certain models, involving overlapping mechanisms of apoptosis, pyroptosis, and necroptosis. In their study, the toxin activated markers characteristic of both caspases and kinases associated with necroptosis (including RIPK3 and MLKL), indicating a more complex and multidimensional cellular response [56]. These findings suggest that OTA cannot be strictly attributed to a single mode of cell death, but rather that its effects are modulated by biological context and environmental conditions.

Against this background, the absence of necrosis markers in our experiments may be interpreted in several ways. First, the concentrations and exposure times employed may have been sufficient to trigger apoptosis but not necrosis. Second, PBMCs, as primary cells, may predominantly undergo programmed cell death, whereas leukemia cell lines, despite failing to undergo apoptosis, also did not shift toward necrosis due to the presence of adaptive mechanisms (e.g., maintenance of ATP levels or activation of antioxidant systems). Third, it cannot be excluded that the detection methods used in our study were less sensitive in identifying early, subtle necrotic changes, which might occur at higher OTA doses or after prolonged incubation.

Our results indicate that OTA selectively induces apoptosis in PBMCs, while leukemia cell lines remain resistant. Such differential sensitivity has important biological and toxicological implications. The susceptibility of PBMCs confirms earlier observations by Moura et al. [57], who showed that OTA exposure in broilers led to marked alterations in leukocyte profiles, including a reduction in lymphocyte and eosinophil percentages and a concurrent increase in heterophils and monocytes [57]. These findings suggest that OTA disrupts immune system balance, thereby promoting immunosuppression. Similar conclusions were drawn by Froquet et al. [58] whose demonstrated that OTA inhibited the proliferation of human hematopoietic progenitors, affecting both the erythroblast and granulocyte-monocyte lineages [58].

Khan et al. [59] investigated how even at low doses OTA exposure in broiler chicks reduced weights of lymphoid organs, a dose-dependent decrease in circulating lymphocytes and heterophils, increased monocytes, and decreased serum concentrations of IgY and IgA levels, indicating pronounced immunosuppression in vivo [59]. In line with this, Khan et al. [60]) demonstrated that even chronic dietary OTA exposure at the recommended maximum level (0.1 mg/kg) decreased IgY and IgA levels, and decreased thymus and bursa size across all exposed groups, highlighting that even regulatory “safe” doses of OTA compromise humoral immunity [60].

Animal studies consistently show negative impact of OTA on hematopoiesis. Hong et al. [24] showed that OTA exposure in mice resulted in long-term inhibition of bone marrow regeneration after irradiation, demonstrating that the toxin reduces hematopoietic reserves and increases susceptibility to additional stressors [24]. Gupta et al. [61] further confirmed that chronic OTA administration in mice decreased platelet counts, hematocrit, spleen mass, and bone marrow progenitor cells [61].

Initially hypothesized as anti-leukemic, OTA instead caused strong apoptosis in PBMCs, while leukemia cell lines were resistant, overturning expectations and highlighting immunotoxic risk. It should be noted, however, that only three leukemia cell lines were investigated in this study. Although representative of distinct hematological malignancies, they may not capture the full heterogeneity of leukemias, and additional models will be necessary to determine whether resistance to OTA is a general feature of malignant hematopoietic cells.

These observations are preliminary and should be interpreted with caution. They are limited to in vitro models, and it remains unclear whether similar effects occur in vivo. While OTA has been linked to hepatocellular carcinoma and renal carcinoma, its potential role in hematological malignancies is largely unexplored [18]. Further studies are essential to elucidate the molecular mechanisms underlying the differential sensitivity between leukemia cells and PBMCs, to assess the contribution of genetic, epigenetic, or metabolic factors, and to evaluate potential interactions with other mycotoxins or environmental stressors. When comparing our findings to animal studies, it is important to consider interspecies differences in OTA metabolism and immune system organization—OTA toxicokinetics vary strongly between species [1,62]. For example, birds possess a unique lymphoid organ, the bursa of Fabricius, and different lymphocyte distributions compared with mammals [63]. Nevertheless, OTA exposure has been repeatedly associated with leukopenia and lymphoid-organ atrophy in avian (e.g., broiler chicks), rodent, and human in vitro models, suggesting a conserved immunotoxic potential across taxa, although absolute sensitivity appears species-dependent [34,59,61]. Overall, OTA’s selective toxicity toward normal immune cells highlights its immunotoxic risks and the need for careful evaluation of its biological effects.

## 4. Materials and Methods

### 4.1. Materials

Dimethyl sulfoxide (DMSO), Tris buffer, Histopaque^®^-1077, RPMI 1640 medium, (S)-(+)-camptothecin (C9911), and OTAfrom *Petromyces albertensis* (catalog no. O1877) were purchased from Sigma-Aldrich Chemical Co. (Sigma-Aldrich Chemical Co., St. Louis, MO, USA). A cell-viability kit containing counting slides and trypan blue dye was obtained from BIO-RAD (Bio-Rad Laboratories, Hercules, CA, USA). Penicillin–streptomycin solution, heat-inactivated fetal bovine serum (FBS), and phosphate-buffered saline (PBS) without calcium or magnesium were supplied by Lonza (Basel, Switzerland). JC-1 dye, 3-(4,5-dimethylthiazol-2-yl)-2,5-diphenyltetrazolium bromide (MTT), Hank’s Balanced Salt Solution (HBSS), and the CellEvent™ Caspase-3/7 Green Flow Cytometry Assay Kit were sourced from Thermo Fisher Scientific (Waltham, MA, USA). The FITC-labeled Annexin V Apoptosis Detection Kit I was obtained from Becton Dickinson (Franklin Lakes, NJ, USA). All other reagents were of molecular-grade quality.

### 4.2. Cellular Material

Peripheral blood mononuclear cells (PBMCs) were obtained from commercially purchased leukocyte–platelet buffy coats supplied by the Regional Centre for Transfusion Medicine in Łódź, Poland. Morning collections (between 8:00 and 10:00 a.m.) were performed on fasting donors who had undergone a medical examination to confirm the absence of cardiovascular disease, allergies, or lipid and carbohydrate metabolism disorders [64]. Within two hours of donation, each buffy coat was carefully diluted and layered at a 1:1 volume ratio onto Histopaque^®^-1077—a sterile solution containing polysucrose (57 g/L) and sodium diatrizoate (90 g/L) with a density of 1.077 g/mL—and centrifuged for 30 min at 400× *g* at room temperature. The isolated PBMC fraction was then washed twice with RPMI 1640 (400× *g*, 10 min, room temperature).

In addition to peripheral blood mononuclear cells, the research also involved cultured human cancer cell lines to evaluate antileukemic effects in vitro. Three well-characterized human leukemia lines were employed: the acute lymphoblastic leukemia line CCRF-CEM (CCL-119), the chronic myelogenous leukemia line K-562 (CCL-243), and the promyelocytic leukemia line HL-60. All three cell lines were obtained from the American Type Culture Collection (ATCC™, Manassas, VA, USA), ensuring authenticated, standardized sources for experimental reproducibility.

### 4.3. Cell Cultures

All cell lines were maintained under identical conditions in RPMI 1640 medium supplemented with 10% (*v*/*v*) FBS, 100 U/mL penicillin, and 100 µg/mL streptomycin sulfate. Cultures were grown in a humidified incubator at 37 °C with 5% CO_2_. For experiments, cells were plated at a density of 3 × 10^6^ cells per well and allowed to equilibrate for 12 h before treatment [65]. Cells were treated with six OTA concentrations: 0.001, 0.01, 0.1, 1, 10, and 100 μM for either 24 or 48 h. (S)-(+)-Camptothecin served as the positive control.

### 4.4. Cell Viability Determination

Cell viability after exposure to OTAwas assessed using two independent methods. The first relied on trypan blue exclusion, performed with a BIO-RAD TC20 automated cell counter (Bio-Rad Laboratories, Hercules, CA, USA) following the manufacturer’s instructions. Results were expressed as the percentage of viable cells relative to the untreated control, defined as 100%. The second method employed an MTT assay. A 0.5 mg/mL MTT solution was added to each sample and incubated at 37 °C for 4 h. After incubation, the solution was carefully removed and the resulting formazan crystals were dissolved in DMSO. Absorbance was measured at 570 nm with background subtraction at 630 nm using a BioTek Synergy HT microplate reader (BioTek Instruments, Winooski, VT, USA). Cell viability was reported as a percentage of the untreated control.

### 4.5. ΔΨM

ΔΨM is one of the most important parameters used for evaluating mitochondrial function—a key indicator of cell health. In this analysis, cell membrane-permeable fluorescent dye—JC-1 (5′,6,6′-tetrachloro-1,1′,3,3′-tetraethylbenzimidazolylcarbocyanine iodide) was used. In the first step, cells were seeded into 96-well black plates with transparent bottoms dedicated to fluorescence measurements (Greiner Bio-One, Kremsmünster, Austria) at a density of 1 × 10^5^ cells/well in 50 μL culture medium and allowed to adhere for 12 h. Next, the cells were incubated with OTA according to the procedure described in the previous point. After treatment, cells were preincubated for 30 min with 5 μM JC-1 dye (prepared in the HBBS) at 37 °C in a CO_2_ (5%) incubator. Finally, cells were centrifuged (300× *g* for 10 min at 22 °C) and washed twice with the HBBS. The fluorescence was measured on a Bio-Tek Synergy HT Microplate Reader (Bio-Tek Instruments, Winooski, VT, USA), with filter pairs of 530 nm/590 nm and 485 nm/538 nm. Results have been presented as a ratio of fluorescence, measured at 530 nm/590 nm to that measured at 485 nm/538 nm (aggregates to monomer fluorescence).

### 4.6. Determining the Activity of Caspase-3/Caspase-7

Caspase-3/7 activity was measured using the CellEvent™ Caspase-3/7 Green Flow Cytometry Assay Kit (Thermo Fisher Scientific, Waltham, MA, USA). Human leukemia cell lines CCRF-CEM, K-562, HL-60, and peripheral blood mononuclear cells (PBMCs) were first exposed to OTAat concentrations ranging from 0.001 to 100 µM for the designated treatment periods. Following incubation, cells were washed twice with ice-cold phosphate-buffered saline (PBS) and resuspended at a density of 1 × 10^5^ cells in 1 mL PBS in flow-cytometry tubes. One microliter of CellEvent™ Caspase-3/7 Green Detection Reagent was added and mixed gently, and samples were incubated for 30 min at 37 °C in a humidified atmosphere containing 5% CO_2_. Subsequently, 1 µL of 1 mM SYTOX™ AADvanced™ (prepared in DMSO) was added and the samples were kept in the dark at room temperature for 5 min. Without further washing or fixation, fluorescence was recorded on a BioTek Synergy HT microplate reader (BioTek Instruments, Winooski, VT, USA) using filter settings of 530/590 nm and 485/538 nm.

### 4.7. Gene Expression Analysis Using Real-Time PCR

Total RNA was isolated from cell pellets using the EXTRACT ME RNA and DNA Kit (BLIRT S.A., Gdańsk, Poland), following the manufacturer’s instructions. The purity and concentration of the extracted RNA were assessed by measuring absorbance at 260 and 280 nm with a Bio-Tek Synergy HT Microplate Reader (Bio-Tek Instruments, Winooski, VT, USA). RNA samples were stored at −30 °C until subsequent analyses. Complementary DNA (cDNA) was synthesized from total RNA using the High-Capacity cDNA Reverse Transcription Kit, with 2 μg of RNA employed as template in a final reaction volume of 10 μL. Gene expression levels were quantified by RT-qPCR employing the TaqMan probe-based detection system using commercially available TaqMan™ Gene Expression Assays (all details regarding the TaqMan™ probes used in our studies are presented in Table 1). RT-qPCR was performed using TaqMan™ Universal Master Mix II (Applied Biosystems™, Thermo Fisher Scientific) and CFX96™ Real-Time PCR Detection System (BIO-RAD Laboratories, Hercules, CA, USA). The RT-qPCR conditions were as follows: 10 min of polymerase activation at 95 °C, followed by 40 cycles of 30 s denaturation at 95 °C and 60 s annealing/extension at 60 °C.

### 4.8. Data Analysis

All experimental measurements were processed using Microsoft Excel (Microsoft Corp., Redmond, WA, USA; version 2021) and are presented as mean ± standard deviation (SD). Statistical analyses were carried out in StatsDirect software, version 2.7.2 (StatsDirect Ltd., Cheshire, UK). Normality of the data distribution was first evaluated with the Shapiro–Wilk test, and homogeneity of variance was assessed with Levene’s test. Differences among groups were then examined by one-way ANOVA followed by Tukey’s post hoc test when assumptions of normality and equal variance were met, or by the Kruskal–Wallis test when those criteria were not satisfied. A *p*-value < 0.05 was considered statistically significant [66,67,68].

## 5. Conclusions

In summary, OTA induced pronounced apoptotic effects in healthy PBMCs but failed to trigger cytotoxic responses in leukemia cell lines CCRF-CEM, K-562, and HL-60. These results highlight the novel finding that OTA selectively comprises normal immune cells while sparing malignant hematopoietic cells. This selective toxicity emphasizes its immunotoxic risk rather than any therapeutic potential. Although limited in vitro models, our study provides a unique comparative analysis that enhances understanding of differential OTA sensitivity and its implications for immune function and hematological health. Given that these findings are based on in vitro models, further investigations—particularly in vivo studies—are required to clarify the molecular mechanisms underlying this differential sensitivity of normal versus malignant hematopoietic cells and to fully assess the immunotoxic risks of OTA exposure.

## Figures and Tables

**Table 1:** Details of the TaqMan™ Gene Expression Assays employed in RT-qPCR experiments.

Gene Symbol	Gene Name	GenBank mRNA	TaqMan™ Gene Expression Assay IDs *	Exon Boundary	Assay Location (Base Position Contained Within the Probe)
18S	Eukaryotic 18S rRNA	X03205.1	Hs99999901_s1	1	604
BAX	BCL2 associated X, apoptosis regulator	AF247393.1	Hs00180269_m1	4–5	370
BCL2	BCL2, apoptosis regulator	BC027258.1	Hs00608023_m1	2–3	977
CASP3	caspase 3	AJ413269.1	Hs00234387_m1	2–3	178
CASP8	caspase 8	AB451282.1	Hs01018151_m1	2–3	413
CASP9	caspase 9	AB015653.1	Hs00962278_m1	3–4	615
APAF1	apoptotic peptidase activating factor 1	AB007873.2	Hs00559441_m1	4–5	674
CHEK1	checkpoint kinase 1	AB451222.1	Hs00967510_g1	9–10	1099
CHEK2	checkpoint kinase 2	AB040105.1	Hs00200485_m1	7–8	908

* Obtained from Applied Biosystems (Thermo Fisher Scientific).

## Data Availability

The original contributions presented in this study are included in the article. Further inquiries can be directed to the corresponding author(s).

## Abbreviations

The following abbreviations are used in this manuscript:

APAF1	Apoptotic protease activating factor 1
ATP	Adenosine triphosphate
BAX	BCL2-associated X protein
BCL-2	B-cell lymphoma 2
DAPI	4′,6-diamidino-2-phenylindole
ΔΨM	Mitochondrial membrane potential
DNA	Deoxyribonucleic acid
ERK1/2	Extracellular signal-regulated kinases 1 and 2
ESR	Erythrocyte sedimentation rate
IgA	Immunoglobulin A
IgY	Immunoglobulin Y
IL	Interleukin
JC-1	5,5′,6,6′-tetrachloro-1,1′,3,3′-tetraethylbenzimidazolocarbocyanine iodide
MLKL	Mixed lineage kinase domain-like pseudokinase
Mcl-1	Induced myeloid leukemia cell differentiation protein
MTT	3-(4,5-dimethylthiazol-2-yl)-2,5-diphenyl tetrazolium bromide
My88	Myleoid differentiation primary response 88
NF-κB	Nuclear factor kappa-light-chain-enhancer of activated B cells
OTA	Ochratoxin A
PANoptosis	Programmed cell death involving apoptosis, pyroptosis, and necroptosis
PBMCs	Peripheral blood mononuclear cells
p53AIP1	p53-regulated apoptosis-inducing protein 1
RIPK3	Receptor-interacting serine/threonine-protein kinase 3
ROS	Reactive oxygen species
RT-qPCR	Reverse transcription quantitative polymerase chain reaction
Socs	Suppressor of cytokine signaling
Stat	Signal Transducer and Activator of Transcription
TLR4	Toll-like receptor 4
TNF-α	Tumor necrosis factor-alpha
TP53	Tumor protein p53
z-VAD-fmk	pancaspase inhibitor
